# The State Medical Association

**Published:** 1879-06

**Authors:** 


The State Medical Association, at its recent session in Rome, Georgia, adopted a resolution providing for the restoration to membership in the Association, of all those whose names had been dropped from the roll for non-payment of dues within the time specified in the By-laws. It was resolved, that all these delinquents be reinstated upon the payment, to the Secretary, of the accumulated dues to date, which amount now to only two dollars.

It is earnestly hoped that all old members will avail themselves of the proposition, and promptly re-unite with the organization by complying with the regulation. The annual assessment is now only one dollar, and none can plead exorbitant dues as excuse for non-payment.

